# Biodegradable Polyurethane Foams Based on Polyols Obtained from Cellulose and Its Hydroxypropyl Derivative

**DOI:** 10.3390/ma17225490

**Published:** 2024-11-10

**Authors:** Renata Lubczak, Małgorzata Kus-Liśkiewicz, Jacek Lubczak, Marzena Szpiłyk, Daniel Broda, Ewa Bobko

**Affiliations:** 1 Department of Organic Chemistry, Faculty of Chemistry, Rzeszów University of Technology, Al. Powstańców Warszawy 6, 35-959 Rzeszów, Poland; rlubczak@prz.edu.pl (R.L.); m.szpilyk@prz.edu.pl (M.S.); 2 Institute of Biotechnology, College of Natural Sciences, University of Rzeszow, Pigonia 1, 35-310 Rzeszow, Poland; mkus@ur.edu.pl (M.K.-L.); dbroda@ur.edu.pl (D.B.); 3 Center for Microelectronics and Nanotechnology, Institute of Materials Engineering, University of Rzeszow, Al. Rejtana 16, 35-959 Rzeszow, Poland; ebobko@ur.edu.pl

**Keywords:** cellulose, (hydroxypropyl)cellulose, glycidol, ethylene carbonate, polyol, polyurethane foams, cellulase, biodegradation

## Abstract

Three methods of cellulose-derived polyol synthesis were elaborated. The suitable substrates were (hydroxypropyl)cellulose or cellulose, which were hydroxyalkylated in reactions with glycidol and ethylene carbonate in triethylene glycol or in water. The products were characterized by IR, ^1^H NMR, and MALDI ToF spectroscopies. For all polyols, IR spectra showed strong bands at 1060 cm^−1^ from the ether group formed upon the ring opening of GL and EC. The polyol obtained from (hydroxypropyl)cellulose in the triethylene glycol solvent was accompanied by oligomeric products of glycol hydroxyalkylation and oligomeric glycidol. The polyol obtained by the hydroxyalkylation of cellulose with glycidol and ethylene carbonate in the water contained units of hydroxyalkylated cellulose and products of hydroxyalkylation of water. The physical properties of the obtained polyols, like density, viscosity, and surface tension, were determined. The polyols were then used to obtain rigid polyurethane foams. The foams have apparent density, water uptake, and polymerization shrinkage similar to classic rigid PUFs. The foams showed advantageous thermal resistance in comparison with classic ones. After thermal exposure, their compressive strength improved. The biodegradation of the obtained materials was tested by a respirometric method in standard soil conditions by the measurement of biological oxygen demand and also using the cellulases or the enzymes responsible for cellulose degradation. It has been found that polyols are totally biodegradable within one month of exposure, while the foams obtained thereof are at least 50% biodegraded in the same conditions. The enzymatic biodegradation of the PUFs by the action of microbial cellulase was confirmed.

## 1. Introduction

Dynamic civilization progress and industrial invasion brought a wide spectrum of new materials that were not available in the biosphere during biological evolution. These chemical materials are applied in industries, buildings, medicine, cosmetics, and food production. However, this progress also resulted in the chemical pollution of the biosphere [1]. All industries suffer from necessary recycling in order to limit the pollution and use of environmentally burdensome side-products [2]. Currently, we are tackling the economic and technical challenges related to the boost of plastic waste and excessive emissions of carbon dioxide. Therefore, the trend of recyclable or biodegradable chemical sources has grown over the last three decades. Within plastic chemistry, the use of fossil hydrocarbons must be limited, and they must be replaced by biodegradable materials and biosphere-available resources [3]. Biopolymers are widely available, environmentally friendly, non-toxic, and biodegradable resources to apply. Biodegradable materials can be divided into four groups, depending on isolation techniques to obtain strictly defined polymers. The first group contains compounds that can be separated from biomass by extraction. The polysaccharides (mostly starch, cellulose, and chitosan) and plant or animal proteins are examples [4]. The second group of biodegradable chemicals includes the polymers produced via the polymerization of monomers available in the biosphere. Polylactide (PLA) is one of such representatives [5,6]. The third group comprises the polymers synthesized by microorganisms, like polymers or copolymers of 3-hydroxybutyrate and/or 3-hydroxyvalerate, and bacterial cellulose. The fourth group includes polymers obtained from fossil hydrocarbons, which are biodegradable, like polyvinyl alcohol (PVA), polyglycolate (PGA), or polycaprolactone (PCL) [7,8].

Polyurethanes are widely used macromolecular materials, mostly fabricated as polyurethane foams (PUFs). Their properties can be easily tuned, and these materials have found industrial, medical, and daily life applications. Properly selected substrates and fabrication conditions enable various materials to be obtained [9,10]. Polyurethanes have good resistance against abrasion, water, atmospheric factors, organic solvents, oils, and grease [11,12]. Polyurethanes constitute 4.6% of plastic production, and PUFs are 80% of it. The PUFs can be semi-elastic, elastic, rigid, or semi-rigid materials of wide use. Rigid PUFs have good mechanical parameters and have found applications as isolation and construction materials. Additionally, they show good adhesion to other materials like glass, metal, wood, and fibers. They are convenient heat and sound isolators, chemically and mechanically resistant, and last but not least, they have a relatively low specific weight [11,12]. Currently, most polyurethanes are synthesized from petrochemical resources, which need to be replaced by biosphere-available substrates [2]. One such substrate is widely available cellulose (CEL). CEL is a basic component of plant tissue that is photosynthesized, and thus, becomes an attractive chemical substrate. On the other hand, CEL is also a degradable material based on the concerted action of light, oxygen, and microorganisms. CEL does not dissolve in water or organic solvents due to the strong inter-chain hydrogen bonds present in its solid form [13].

CEL was not used as a suitable polyol for the synthesis of polyurethanes or PUFs. However, it was used as a material filler and introduced as an additive powder in the foaming process of polyol [14,15]. For rigid PUFs, lignin (9–36%) was also added into fossil-derived polyol, followed by foaming [16]. There are also known polyurethane foams obtained using tannin extracts [17].

**Scheme 1:**
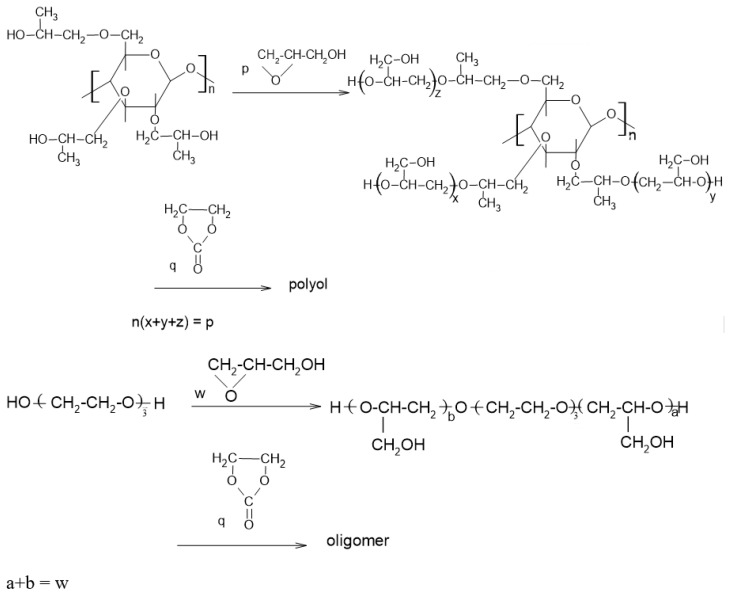
Synthesis of polyols from HPC in TEG.

Although CEL is insoluble, the liquefaction of CEL in glycerol was applied, and the obtained polyol was mixed with up to 70% of petrochemical-derived polyol [18]. Attempts have also been made to incorporate CEL fibers into a polyol additive with its further conversion into PUF. The obtained PUFs had better mechanical properties enhanced, thermal stability, and diminished flammability [19,20,21,22,23]. Recently, the methods of CEL liquefaction were improved, which enabled a series of rigid PUFs to be obtained [24,25,26]. The hydroxypropyl derivative of CEL (HPC) was used with a high degree of hydroxypropyl residues (three-on-one CEL mer). Polyol was obtained from this material by its reaction with glycidol (GL) in triethylene glycol (TEG) and further functionalization with ethylene carbonate (EC) according to Scheme 1.

**Scheme 2:**
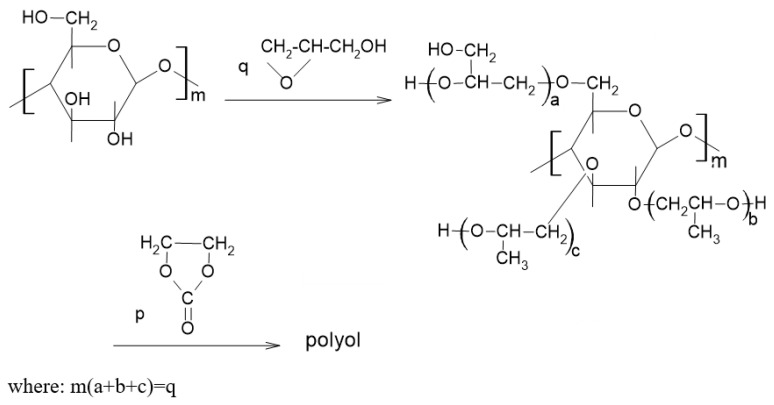
Synthesis of polyol from CEL.

Another successful method to obtain CEL-based PUFs was elaborated using partially hydrolyzed CEL (CNC) [25]. Acidic hydrolysis resulted in the partial dissolving of CNC due to the shortening of CEL chains. CNC was hydroxyalkylated with GL and then with EC [25], which is illustrated in Scheme 2.

The hydroxyalkylation of CEL is also possible when CEL is preliminarily swelled in water and treated with GL and then with EC [26].

Here, we describe the structure and physical properties of CEL-based polyols obtained in all three methods and the polyurethane foam obtained from these polyols. The novelty of the presented research is to test the biodegradation of obtained materials in standard soil conditions as well as using commercially available cellulases.

## 2. Experimental

### 2.1. Materials

The following materials were used in this work: (hydroxypropyl)cellulose (HPC powder, 20 mesh particle size, 99% purity, Merck, Darmstadt, Germany), cellulose (CEL, powder, 20 mesh particle size, 99% purity, Sigma-Aldrich, Taufkirchen, Germany), glycidol (GL, pure, Sigma-Aldrich, Taufkirchen, Germany), potassium carbonate (pure, POCH, Gliwice, Poland), ethylene carbonate (EC pure, Fluka, Buchs, Switzerland), triethylene glycol (TEG, Merck, Darmstadt, Germany), triethylamine (TEA, pure, Fluka, Buchs, Switzerland), surfactant Silicon L-6900 (pure, Momentive, Wilton, CT, USA), and polymeric diphenylmethane 4,4′–diisocyanate (pMDI, Merck, Darmstadt, Germany).

### 2.2. Syntheses

#### 2.2.1. Polyol from HPC

In total, 15.1 g GL and 26.64 g TEG were placed in a 250 cm^3^ round-bottomed three-neck flask equipped with a mechanical stirrer, reflux condenser, and thermometer. HPC was added stepwise with vigorous stirring; the mixture was gently heated until HPC dissolved. Then, the mixture was heated to 150 °C for 15 min, and the temperature was raised gradually up to 200 °C in order to observe the complete reaction of GL. Then, the mixture was cooled down to 50 °C; 54 g EC was added; and afterward, 0.20 g K_2_CO_3_ catalyst was added. The mixture was heated at 170–175 °C for 7 h. The reaction was considered complete based on the mass balance due to the consumption of EC and analytical determination of EC in the current sample of the mixture. The obtained product was dark brown resin, HPC-TEG-GL-EC [24].

#### 2.2.2. Hydrolysis of Cellulose

The hydrolysis of cellulose (CNC) was performed, as exemplified in [19,20,21]. To be specific, 500 cm^3^ of 64% sulfuric(VI) acid was placed in a 1500 cm^3^ three-necked round bottom flask equipped with a reflux condenser, mechanical stirrer, and thermometer. Cellulose (50 g) was suspended in a vigorously stirred solvent, and the mixture was heated at 45 °C for 2 h. The contents of the flask were then poured into 750 cm^3^ of water to stop the hydrolysis. The colloidal solution was neutralized with 50% sodium hydroxide in the presence of phenolphthalein. The neutralized mixture was centrifuged to obtain colloid, which was washed with water until pH = 7. The resulting cellulose pulp was dried at 90 °C for 6 h. Overall, 35 g of dry hydrolysate was obtained, which was then ground into powder.

#### 2.2.3. Polyol from CNC

In total, 29.6 g GL (0.40 mol) was placed in a 100 cm^3^ round-bottomed three-neck flask equipped with a mechanical stirrer, reflux condenser, and thermometer. Hydrolyzed cellulose (4.86 g, corresponding to 0.03 mol of mers) was added stepwise with vigorous stirring. The mixture was heated to 155 °C, at which an exothermic effect was noticed, resulting in an increase in temperature to 190 °C. Furthermore, the mixture was kept at 180 °C for 18 h. Then, the mixture cooled down to 80 °C, followed by the addition of EC (35.2 g; 0.40 mol) and potassium carbonate (0.15 g; 0.001 mol) as a catalyst. The mixture was heated at 165 °C for 6 h. The dark brown resin of CNC-GL-EC was obtained [25].

#### 2.2.4. Polyol from CEL

In total, 70.3 g glycidol (0.95 mol) and 15 g of water were placed in a 250 cm^3^ round-bottomed three-neck flask equipped with a mechanical stirrer, reflux condenser, and thermometer. Next, 8.1 g (0.05 mole of mers) CEL was added while vigorously stirring. The mixture was gently heated at 180 °C for 30 h until all CEL was dissolved. Then, the mixture was cooled down to 80 °C, and 70.4 g (0.8 mol) EC and 0.50 g K_2_CO_3_ were added. The mixture was heated at 180 °C until the completion of the reaction (ca 8 h). The dark brown resin of CEL-H_2_O-GL-EC was obtained [26].

### 2.3. Analytical Methods

The molecular weight (number-averaged and weight-averaged) and dispersity of the HPC, CNC, and polyols were determined by gel permeation chromatography (GPC) with a set composed of Agilent Technologies 1260 Iso Pump Infinity II, TSKgel HHR-H Guard and TSKgel GMHH-M columns, Shodex RI-71 differential refractometer (Showa Denko, New York, NY, USA), and OmniSEC, v4.2 software (Agilent Technologies, Santa Clara, CA, USA). The measurements required the following conditions: 22 °C temperature, 1 cm^3^/min volume flow of eluent, 20 mL volume of the inlet chamber, 4–5 mg/cm^3^ polymer concentration, 30 min analysis time, N,N-dimethylformamide eluent, and polystyrene as a calibration reference.

The size of CEL and CNC was determined by a Dynamic Light Scattering method with a Zetasizer Nano ZS, Malvern instrument ( Malvern, Worcestershire, UK). The samples of the polymer were dispersed by 15 min ultrasonification in ethylene glycol (c = 1 mg/mL) at 25 °C.

The reaction of cellulose with GL was monitored by epoxide number determination using hydrochloric acid in dioxane [27]. The progress of hydroxyalkylation with EC was determined using the barium hydroxide method described in [28]. Finally, the hydroxyl number (HN) of polyol was determined by acylation with acetate anhydride in xylene. The excess of anhydride was titrated with 1.5 M NaOH_aq_ in the presence of phenolphtalein [29]. The ^1^H-NMR spectra of reagents were recorded at 500 MHz Bruker UltraShield in DMSO-d_6_ with hexamethyldisiloxane as the internal standard. IR spectra were registered on an ALPHA FT-IR BRUKER spectrometer in KBr pellets or by the ATR technique. The MALDI ToF (Matrix-Assisted Laser Desorption Ionization Time of Flight) spectra of polyols were obtained on a Voyager-Elite Perceptive Biosystems (US) mass spectrometer working in the linear mode with delayed ion extraction, equipped with a nitrogen laser working at 352 nm. The method of laser desorption from silver nanoparticles (AgNPET LDI MS) was applied [30]. The observed peaks corresponded to the molecular ions Na^+^ and K^+^ (from the catalyst). The samples were diluted with water at 0.5 mg/mL.

### 2.4. Physical Properties of Polyol

The density, viscosity, and surface tension of polyol were determined with a picnometer, Höppler viscometer (typ BHZ, prod. Prüfgeratewerk, Germany), and by the detaching ring method, respectively.

### 2.5. Obtaining the Polyurethane Foams

Three polyols were used to obtain polyurethane foams (PUF), namely those obtained from the following: (i) HPC and GL + EC in TEG, (ii) CNC and GL + EC, and (iii) CEL and GL and EC in water.

The foaming of polyol was performed at 500 cm^3^ cups at room temperature. The foams were prepared from 10 g of polyol, to which 0.27–0.88 g of the surfactant (Silicon L-6900) and 0.03–0.88 g of TEA as the catalyst and water (2–3%) as the blowing agent was added. After homogenization, the polymeric diphenylmethane 4,4′-diisocyanate was added. The commercial isocyanate containing 30% of tri-functional isocyanates was used. The mixture was vigorously stirred until creaming began. The samples for further study were cut from the obtained foam.

### 2.6. Properties of Foams

The apparent density [31], water uptake [32], dimensional stability in 150 °C temperature [33], heat conductance coefficient (IZOMET 2104, Bratislava, Slovakia), and compressive strength [34] of PUFs with flame retardants were measured. The thermal resistance of modified foams was determined both by static and dynamic methods. In the static method, the foams were heated at 150, 175, and 200 °C with the continuous measurement of mass loss and determination of mechanical properties before and after heat exposure. In the dynamic method, the thermal analyses of foams were performed in a ceramic crucible at 20–600 °C temperature range, about 100 mg sample, under air atmosphere with Thermobalance TGA/DSC 1 derivatograph, Mettler, with 10 °C/min heating rate. Differential scanning calorimetry (DSC) studies were performed with DSC822^e^Mettler Toledo calorimeter at 20 ÷ 300 °C temperature range, 10 deg/min heating rate, 10 ÷ 20 mg samples under nitrogen atmosphere. Topological pictures of PUFs were also recorded for cross-sections of PUF samples cut in a direction perpendicular to growing. The pictures were taken with 60-fold enlargement and analyzed with MORPHOLOGI G3 (prod. Malvern) using OPTAVIEW—IS v4.0 software.

### 2.7. Biodegradation of Polyol and PUF

The biodegradation of polyol and the PUF obtained from it was tested by the use of the OxiTop Control S6 instrument (WTW-Xylem, Rye Brook, NY, USA). The respirometric method was used to measure the oxygen demand necessary for the aerobic biodegradation of polymeric materials in soil. The measurement of consumed oxygen was presented using the value of biochemical oxygen demand (BOD), which is the number of milligrams of captured oxygen per mass unit of the tested polyurethane material. The instrument was composed of six 510 cm^3^ glass bottles equipped with rubber quivers and measuring heads, which were used to measure BOD. They allowed us to measure the pressure in the range of 500 to 1350 hPa with an accuracy of 1% at a temperature of 5 to 50 °C. The communication between the measuring heads and the user was performed with Achat OC110 computer software (WTW-Xylem, Rye Brook, NY, USA), which was applied to interpret the obtained measurement results.

The biodegradation tests were performed according to the norm [35]. For a biodegradation test, sieved and dried gardening soil was used with the following parameters: 5% humidity (according to ISO 11274 [36]), pH 6 (acc. to ISO 10390 [37]), and particle diameters < 2 nm. The measurement was carried out in a system consisting of 200 mg of the tested sample (polyol or foam), 200 g of soil, and 100 g of distilled water. The samples were homogenized in bottled rubber quivers containing two pastilles of solid NaOH, mounted and sealed with measuring heads for six samples. The set was incubated at 20 ± 0.2 °C for 28 days. The current oxygen consumption was determined within 2–3 day intervals for the samples and two references, positive and negative plus blank, which was for soil and water only. The starch was used as a positive sample, while polyethylene was a negative sample.

BOD was determined for every sample, taking into account the fact that the BOD of the tested system was reduced by the BOD of the soil and concentration of the tested compound in the soil using the following formula:

(1) $$BODs=\frac{{BOD}_{x}-{BOD}_{g}}{c}$$

where S—length of measurement (in days); BOD_S_—biochemical oxygen demand of the analyzed sample within S days (mg/L); BODx—biochemical oxygen demand of the measured system (bottle with sample and soil) (mg/L); BODg—biochemical oxygen demand of soil without a sample (mg/L); c—sample concentration in the tested system (mg/L).

The degree of biodegradation of the polyol or foam was determined using the formula:

(2) $$D_{t}=\frac{{BOD}_{S}}{TOD}\cdot 100\%$$

where Dt—biodegradation degree of sample (%); TOD—theoretical oxygen demand (mg/L).

The theoretical oxygen demand was calculated using the formula given for the norm. It has been assumed that in oxygen conditions, carbon is converted into carbon dioxide, hydrogen into H_2_O, and nitrogen into NH_3_. For the compound with a known C, H, N, and O percentage alongside the total mass of the sample, the TOD value can be calculated from the following equation:

(3) $$TOD=\frac{16\cdot [2C+0.5\cdot \left(H-3N\right)-O]}{m}$$

where C, H, N, O—mass fractions in biodegraded material; m—the sample mass of material (g).

### 2.8. Microbial Enzymatic Biodegradation

Biodegradation studies were performed with at least three repetitions. The solid form of PUFs was cut into small pieces (0.5 cm dimension) and transferred into the 2 mL microtubes with 1 mL of citrate buffer (pH 5.0). Samples were inoculated with 1 mg of a specific enzyme in a 0.2 mL buffer solution. The samples with cellulase from *Aspergillus niger* (Apollo Scientific, BIC2224 Whitefield Road, Bredbury, UK) were incubated at 37 °C, and samples with an enzyme blend (SIGMA, SAE0020, Darmstadt, Germany) were incubated at 50 °C for 3 days. After 24, 48, and 72 h, an aliquot of the samples was taken for glucose (cellobiose) concentration determination with the DNS method (a colorimetric technique that consists of a redox reaction between 3,5-dinitrosalicylic acid and the reducing sugars present in the sample) [38]. Scanning electron microscope (SEM) characterization was used to determine the morphology of polymer pieces. SEM was realized with a Phenom Pro Thermo Fisher Scientific Microscope (AZONANO, Eindhoven, Netherlands), using the “Charge reduction mode” technique, at an accelerating voltage of 10 kV. Several measurements were made for each sample without coating them with a layer of conductive material. The analyses were performed using different magnifications (200–1000×), while the figure later in the work shows the results for 300× magnification. Negative controls were samples without any incubations or samples immersed in the citrate buffer but without enzyme treatment.

## 3. Results and Discussion

### 3.1. Synthesis and Properties of Polyols

HPC is better soluble both in water and organic solvents than CEL. According to the literature, HPC is relatively soluble in polyhydroxy alcohols, like TEG. We chose the HPC with three hydroxypropyl substituents per mer, as was determined by the elemental analysis of HPC (calculated: % C 53.57, % H 8.44; found: % C 53.57, % H 8.33). The product after the conversion of HPC by GL was semi-solid resin, not miscible with isocyanate. In order to liquefy this semi-product, it was further converted by its reaction with EC using its minimum amount, i.e., such that the product obtained was a resin with a viscosity, allowing it to be mixed with isocyanate. The product contained 6.6 mass % of HPC mers in the obtained polyol.

**Figure 1:**
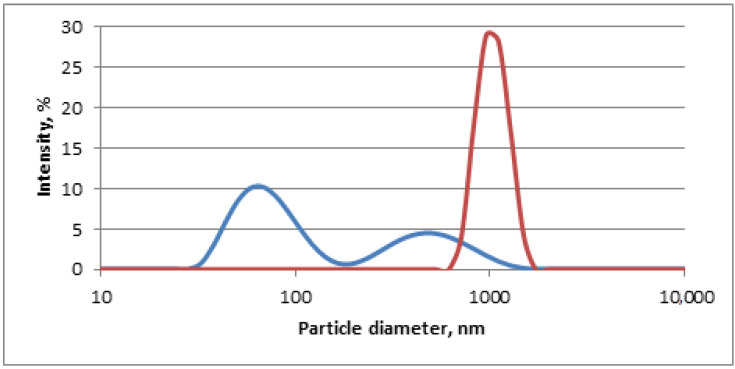
Cellulose particle size distribution before (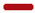) and after (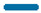) hydrolysis obtained by the DLS method.

In another protocol, the hydrolyzed CEL (CNC) was used as a substrate obtained previously by hydrolysis in sulfuric acid [39,40,41]. The size distribution of particles determined by DLS showed that non-hydrolyzed cellulose had particles within the 200–1200 nm range (Figure 1, red line), while the size was reduced upon hydrolysis mostly to 50–200 nm (Figure 1, blue line). Unassociated particles and/or those that remained untouched by hydrolysis formed a fraction of 200–1200 nm size. The average molecular mass of CNC was determined by GPC, which confirmed the presence of two fractions, namely 62% of *M*_n_ = 9855 u and 38% of *M*_n_ = 1125 u. The dispersion index was 2.1 and 1.3, respectively. During the synthesis, it was noticed that CNC had reacted already at 155 °C, as suggested by the exothermic increase in temperature, while part of CNC remained undissolved in the reaction mixture. Further heating at 180 °C resulted in the complete dissolving of CNC and gave a semi-product, which was then hydroxyalkylated by its reaction with EC (Scheme 2). The contribution of CNC mers in the obtained polyol was 9.3 mas %.

In the third method, the CEL of the particle size within 800–1200 nm (cf. Figure 1, red line) was used.

The contribution of CEL mers in the obtained polyol was 6.3 mass.

**Figure 2:**
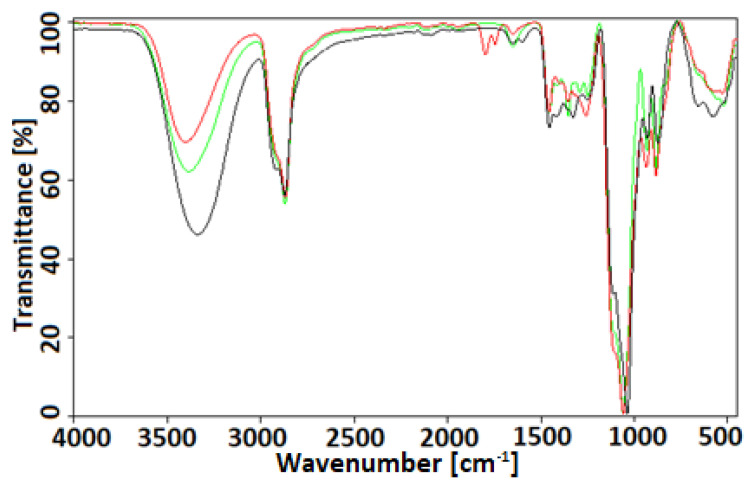
IR spectrum of polyols HPC-TEG-GL-EC (green line), CNC-GL-EC (red line), and CEL-H_2_O-GL-EC (black line).

The progress of the reaction was monitored by IR and ^1^H-NMR spectroscopies and MALDI—ToF spectrometry. The IR spectra of all polyols are very similar (Figure 2). The strong band at 1060 cm^−1^ was attributed to ether groups, which were formed in the ring-opening reactions of GL and EC. The band centered at 2900 cm^−1^, corresponding to the hydroxyl bond stretching vibration in HPC and CEL [24,25], increased due to the formation of oxyalkylene chains accompanied by the appearance of methylene and methine groups resulting from GL and EC ring opening. The deformation vibration bands of these groups were found at 1440–1480 and 1340 cm^−1^, respectively, and partially overlapped with the hydroxyl group deformation band.

**Figure 3:**
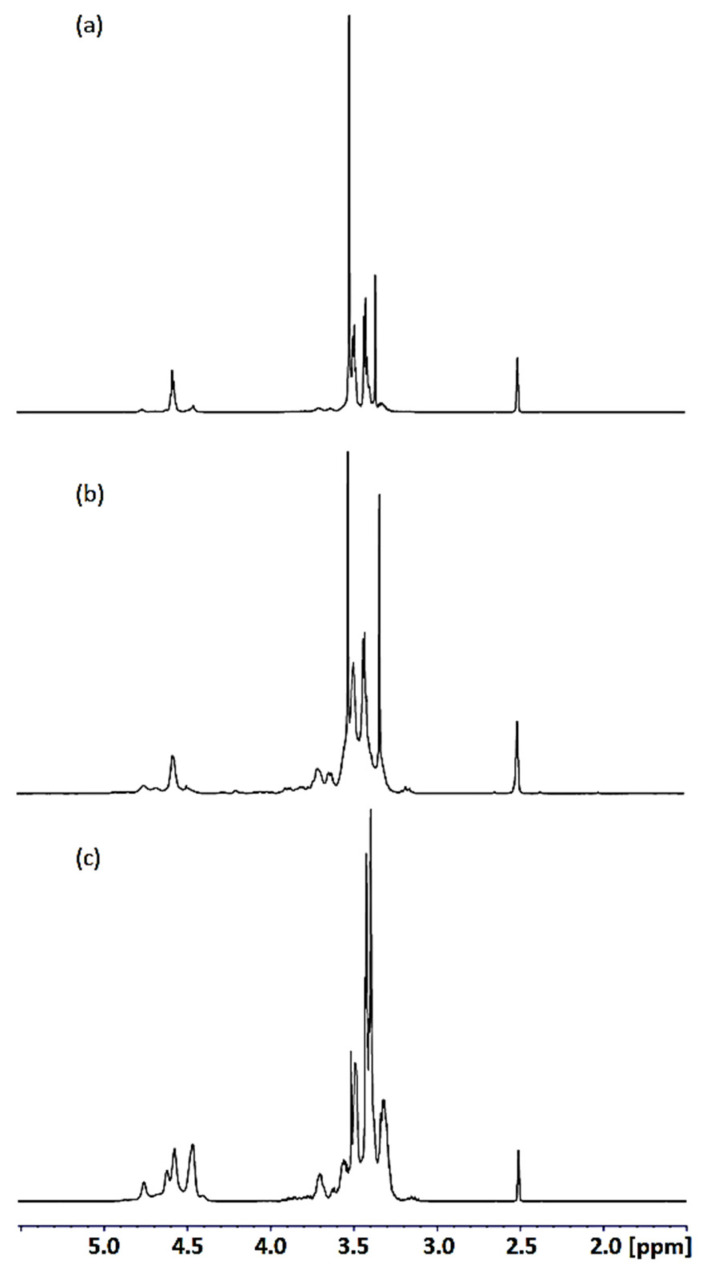
^1^H-NMR spectrum of polyols HPC-TEG-GL-EC (**a**), CNC-GL-EC (**b**), and CEL-H_2_O-GL-EC (**c**).

The ^1^H-NMR spectra of polyols are shown in Figure 3. The resonances within 3.30–3.43 ppm belong to methylene and methine protons, which were present in the following: HPC, CNC, and CEL. Additional methine and methylene proton resonances in the 3.35–3.50 ppm region evidenced the presence of products that reacted with these biopolymers with hydroxyalkylating agents. The hydroxyl proton resonances were observed within 4.4–4.8 ppm. The evidence for their presence in this range is the disappearance of these signals after adding D_2_O to the system.

**Scheme 3:**
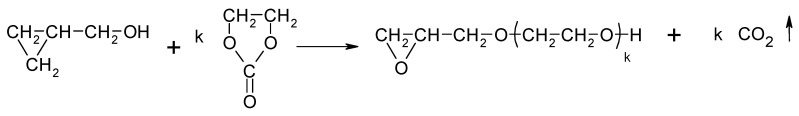
Hydroxyalkylation of GL with EC.

**Scheme 4:**
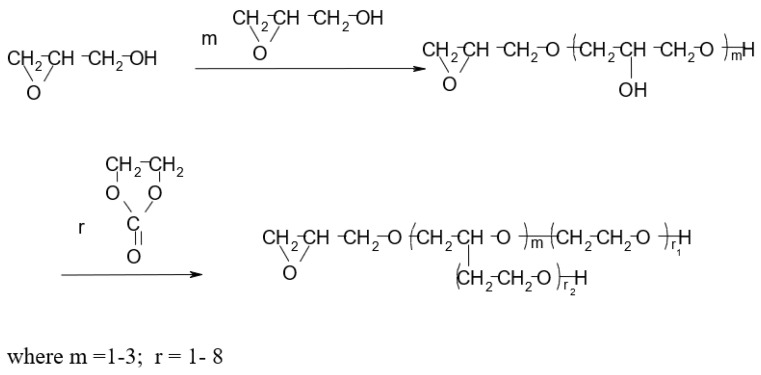
Reactions of oligomeric GL with EC.

To analyze low-molecular-weight products, the MALDI-ToF method was applied, which was already demonstrated to detect the products of the oligomerization of GL and hydroxyalkylation of GL with EC, but not products of the hydroxyalkylation of HPC, CNC, or CEL. The MALDI-ToF spectrometry of polyols obtained from CNC and CEL showed the presence of products of the oxyalkylation of GL by EC (Scheme 3, Table 1, entries 2, 3, 7, 9, 12, 15, 18, 21; Table 2, entries 6, 8), as well as products of reaction between oligomers of GL with EC (Scheme 4, Table 1, entries 10, 11, 13, 14, 16, 17, 19, 20, 22, 23, 25, 26–28, 30, 31, 33, 35; Table 2, entries 3, 6, 8, 10, 12–24, 26–30), the latter of which was also present in polyol obtained from HPC [24].

**Scheme 5:**
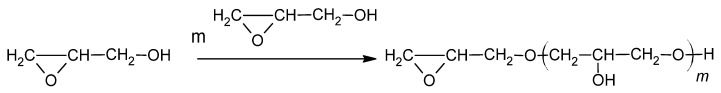
Oligomerization of GL.

It was also found that products of GL oligomerization were present in the polyol obtained from HPC (Scheme 5) [24].

In the case of polyol synthesized in TEG, the products of TEG hydroxyalkylation with GL were also found (Scheme 1) [24].

**Scheme 6:**
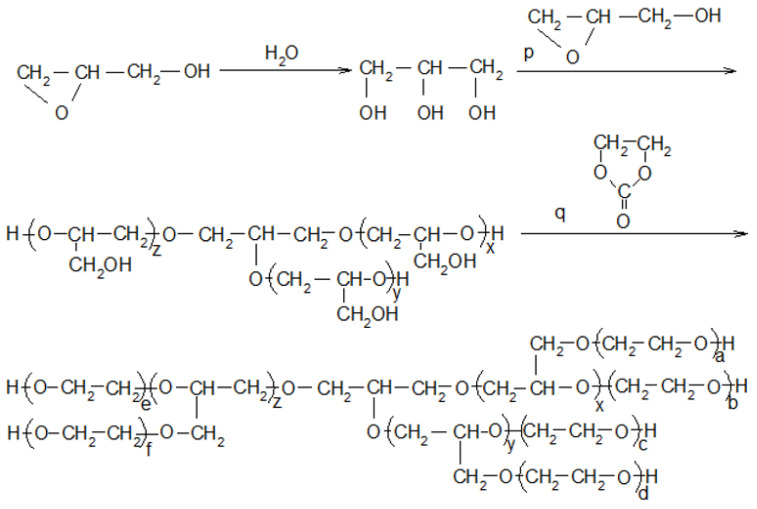
Reaction of hydroxyalkylation in water.

MALDI-ToF results indicated that the hydroxyalkylation of CEL in water resulted in the formation of glicerol by the reaction of GL with water and products of consecutive reactions with glycidol and EC (Scheme 6, Table 1, entries 4–20, 22–31):

The molecular mass of polyols obtained from the HPC and CNC performers using the GPC method indicated the presence of two fractions. In the case of HPC, the high *M*_n_ fraction was 7 mass% (Table 3). The hydroxyalkylated HPC is the dominating component in this fraction considering the molecular mass, while the remaining 93 mass% is the fraction containing oligomers formed in the reaction between TEG with GL and oligomers of GL. The dispersion index of both fractions was within the range of 1.4–1.6. The fraction of polyol 99,860 indicated the products of the reaction of GL and EC with HPC, taking into account that the initial molecular mass of HPC was *M*_n_ = 54,535 u, as determined by GPC.

The polyol obtained from CNC showed lower *M*_n_ than that from HPC, resulting in its higher reactivity. Within this, the fraction *M*_n_ = 9855 prevails (62 mass%) while the number-average molecular weight of CNC before the reaction was 1425 u. For the second fraction corresponding to oligomeric products, *M*_n_ = 1125, and u remained at 38%. The polydispersity indexes of these two fractions were 2.1 and 1.3, respectively.

The polyol obtained from CEL showed only one fraction of relatively low *M*_n_ due to the presence of oligomeric products from the reaction between GL and water and products of cellulose degradation that could be formed due to long (38 h) hydroxyalkylation at 180 °C.

Obtained polyols have various densities (Table 4). The polyol obtained in the TEG medium has the lowest density, which was attributed to the less dense space-packing of polymer chains accompanied by the presence of oligomers formed by TEG hydoxyalkylation. The higher density of polyols obtained from CNC and CEL is probably due to prolonged heating at the synthetic stage, which resulted in the condensation of hydroxyalkylated chains. Polyols also showed various viscosity. The polyol HPC-GL-TEG-EC had the highest viscosity due to the use of a longer oxyalkylene substrate, namely TEG and HPC. The lower viscosity of polyols obtained from CNC and CEL was caused by the presence of shorter cellulose chains, which degraded as a result of hydrolysis or the use of water in the case of CEL. It has been noticed that obtained polyols have comparable surface tension. Low-surface tension is believed to be a beneficial property considering such a polyol as a substrate for foaming towards polyurethane foam (PUF). A higher value, as in the case of obtained polyols, requires the use of a larger amount of surfactants during foaming. Generally, the differences in physical properties of polyols resulted from the variable molar ration of reagents and temperature of synthesis. Obtained polyols have hydroxyl numbers within 473–688 mg KOH/g, which suggests that they can be useful substrates to obtain rigid PUFs thereof.

### 3.2. Synthesis and Properties of Polyurethane Foams

For foaming composition optimization, the amount of pMDI and other components related to the mass of polyol used varied. Table 5 shows the optimal compositions of foamed compositions. We aimed at rigid PUF with small, regular pores and found that the best PUF was obtained using an estimated amount of pMDI in such a way that the molar ratio of the isocyanate group to hydroxyl group (–NCO/–OH, so-called isocyanate coefficient) was 1.5 for the PUF obtained for polyol from HPC and 1.0 from other polyols. The optimum amount of water was 2 mass%; more water (3 mass%) resulted in fragile PUF with large pores, while less water led to under-foamed PUF with a high apparent density, above 100 kg/m^3^. The foaming composition with HPC and CEL substrates required 0.2–0.3 mass% of the catalyst. A lower amount of the catalyst led to PUF with large, irregular pores, while a catalyst of more than 0.3% gave low-foamed PUF with high apparent density. In the case of the composition with CNC, more catalyst was necessary, namely 1.3 g/100 g polyol.

The optimized surfactant amount was within 3.1–3.9 mass%, except for the composition obtained from HPC-TEG-GL-EC, where it was 8.8 g/100 g polyol due to the high viscosity of the polyol.

For optimized compositions, the creaming time was within 32–129 s, while the growing time was within 32–157 s, depending on the kind of polyol used. The longest growing time was needed to form PUF from polyol based on CEL. The compositions dried immediately after growth.

The following properties of optimized PUFs were determined: apparent density, water uptake (by volume), dimensional stability, thermal conductivity coefficient, heat resistance, compressive strength, and glass transition temperature (Table 6). The apparent density of obtained PUFs was within the 58–73 kg/m^3^ limits. Water uptake after 24 h exposure was merely 2–5%, which suggested the presence of closed pores in materials. The highest water uptake value was found for PUF obtained from CEL in water, namely 6.56%. Low values of water uptake of PUF are beneficial for the prospective use of these materials as heat isolators working in high-humidity conditions. The thermal conductivity coefficient of obtained PUFs was comparable and fell within 0.0338–0.0364 W/m·K, i.e., they are in the upper limit of rigid PUF.

The obtained PUFs had good dimensional stability; the PUFs obtained from CNC and CEL polyols shrunk by a maximum of 5.1% upon heating at 150 °C, while PUF from HPC-TEG-GL-EC showed shrinkage and expansion in perpendicular directions.

The density was determined with 2.3% accuracy.

The standard deviation in the case of absorption of water and dimensional stability did not exceed 0.7% and 1.5%, respectively.

The thermal conductivity coefficient was determined with 0.1% accuracy.

**Figure 4:**
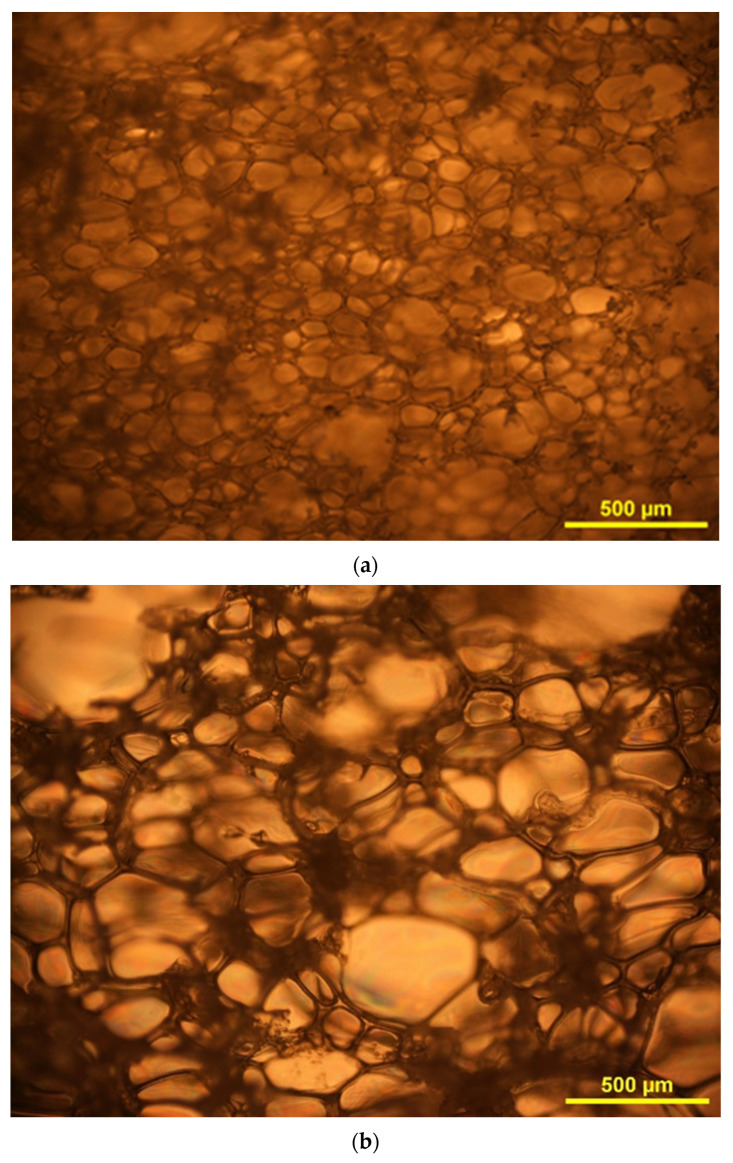

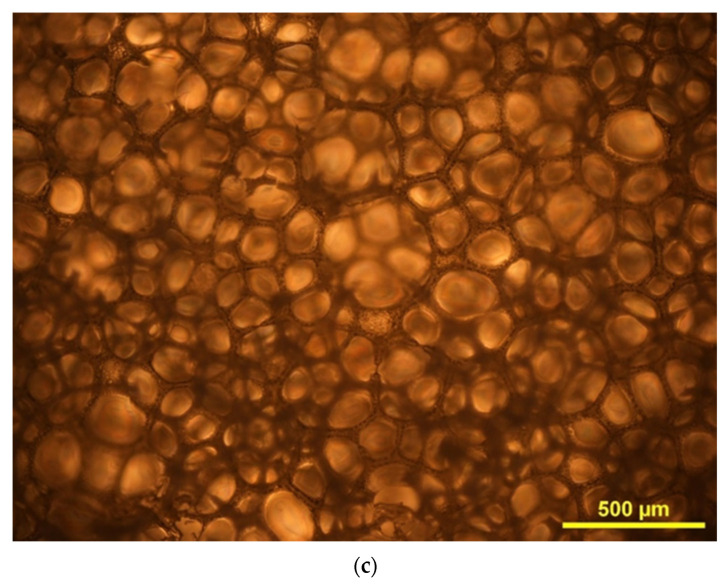
Optical microscopy images of foam obtained from polyols HPC-TEG-GL-EC (**a**), CNC-GL-EC (**b**), and CEL-H_2_O-GL-EC (**c**).

The average pore size of the obtained PUFs was estimated by taking pictures with an OPTA-TECH No.110652 microscope using OptaView-IS v4.0 software. The PUFs obtained from HPC and CEL showed oval pores with longer and shorter axes equal to 140 µm and 90–110 µm, respectively (Table 7, Figure 4). Thus, the pores were smaller than in other rigid PUFs; for instance, the one obtained from CNC-GL-EC had larger pores (cf. Figure 4a,c with Figure 4b). In terms of numbers, rigid PUFs usually have a 200–600 μm pore diameter [42], such as the case of PUF obtained from CNC-GL-EC. Generally, it is rare to obtain rigid PUFs with a pore size below 150 μm [43].

**Figure 5:**
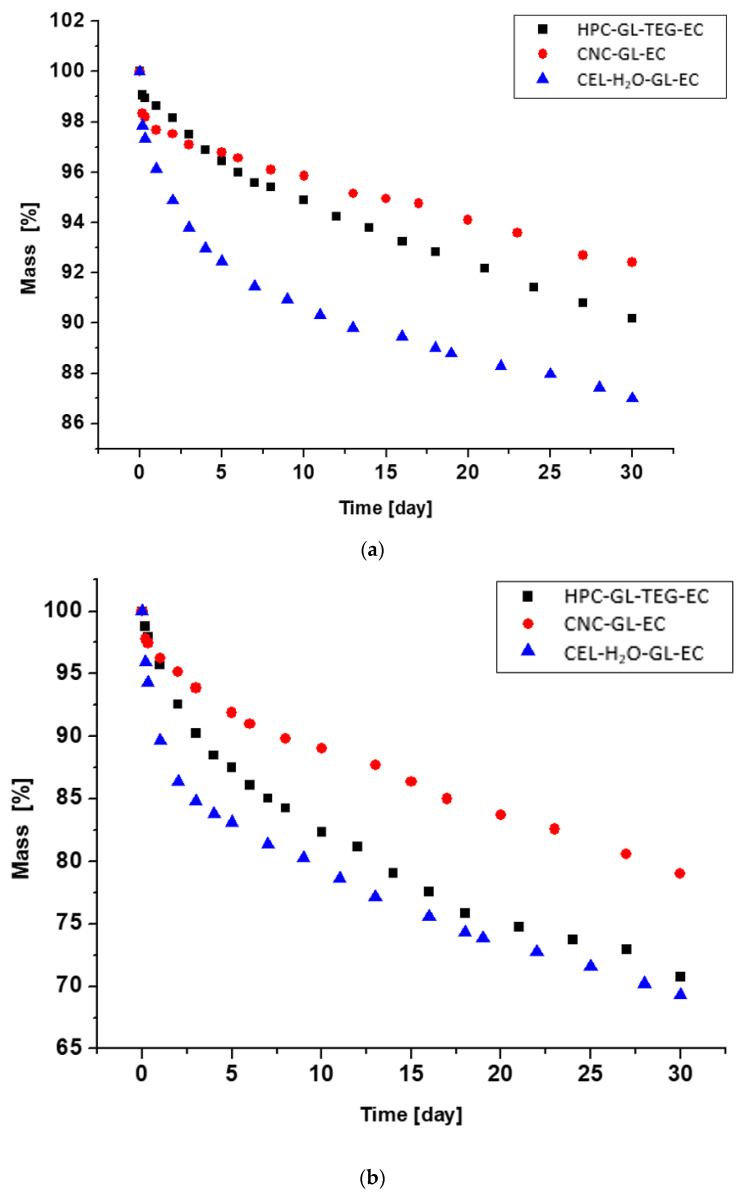
Thermal stability of polyurethane foams at temperatures of 150 °C (**a**) and 175 °C (**b**).

The thermal resistance tests of foam (Table 8) conducted using the static method showed that a large mass loss of PUF samples was observed within the first 2 days of exposure (Figure 5). Mass loss is related to physical processes (like moisture removal and porophore diffusion) and chemical processes named generally as the thermal decomposition of PUF.

The symbol of polyol from which PUF was obtained is given as an insert.

It was found that PUFs obtained from HPC and CNC were thermally resistant at 150 °C; at that temperature, the mass losses were as follows: 9.8 and 7.6%, respectively, upon one month of exposure. The PUF obtained from polyol CEL-H_2_O-GL-EC was slightly less resistant (13% mass loss). Furthermore, the PUF obtained from CNC-GL-EC polyol lost merely 21% upon exposure at 175 °C. The latter is comparable with those thermo-resistant PUFs containing incorporated 1,3,5-triazine, perhydrotriazine, purine, and carbazole rings [44,45]. However, obtained PUFs were not resistant at 200 °C; their mass loss was within 44.5–46.2%. Moreover, the PUF obtained from the CEL-H_2_O-GL-EC polyol was not very thermally resistant. The PUF showed 30.7% mass loss at 175 °C and 46.2% at 200 °C after 1 month of thermal exposure. The reason for such behavior was attributed to pressure jumps within large pores (Figure 4b).

The compressive strength of PUFs was within 0.212–0.279 MPa, which is typical for classic rigid PUFs (Table 8). It is noteworthy that after 30 days of thermal exposure at 150 °C, the PUFs obtained from HPC-TEG-GL-EC and CEL-H_2_O-GL-EC increased by about 21–25%, while PUF obtained from CNC-GL-EC polyol showed a 53% increase. Thermal exposure at 175 °C led to doubling the compressive strength in comparison to non-annealed PUF. The increase in compressive strength upon thermal exposure was due to additional crosslinking at 150 °C. At a higher temperature, the degradation started, and the structure of PUF changed with the partial graphitization and formation of stiff material with improved compression strength (0.725–0.519; MPa at 200 °C), despite severe mass loss (44.5–46.2%, cf. Table 8, columns 4 and 8).

**Figure 6:**
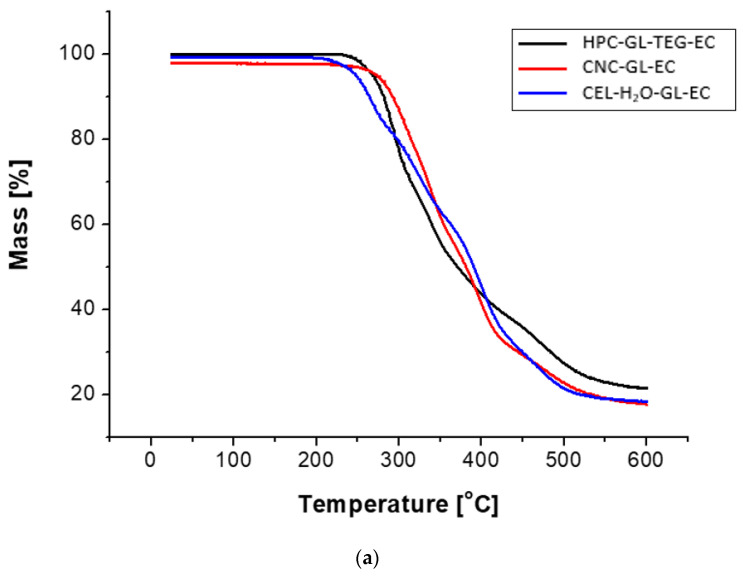

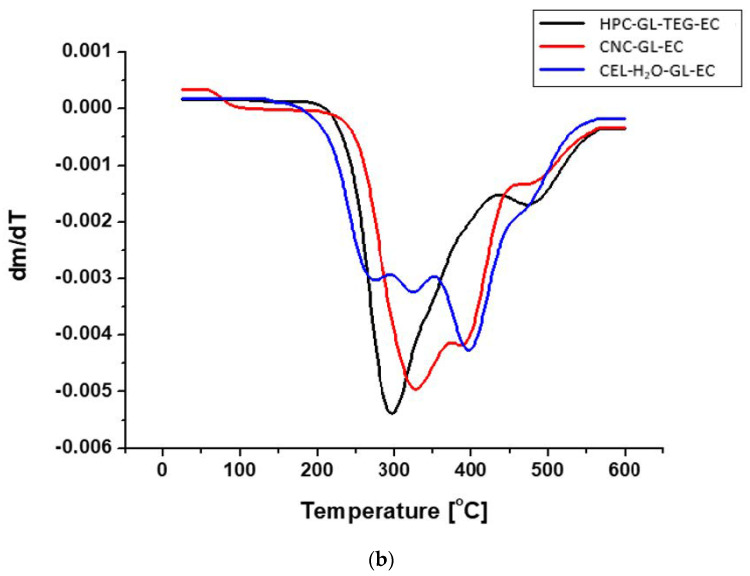
Thermal analysis of foam obtained from polyols: mass change as a function of temperature (**a**); differential mass change as a function of temperature (**b**). The symbol of polyol from which PUF was obtained is given in the insert.

Thermogravimetric analysis in the dynamic mode confirmed that the obtained PUFs possess considerable thermal resistance (Figure 6). The 5% mass loss was observed within 245–274 °C, while maximal mass loss was within 293–397 °C **(**Table 9). The mass of the sample that remained after total decomposition was equal to 18–25% of the initial sample mass (Figure 6a). DSC analysis indicated that glass transition of PUFs was within 92–97 °C, which is the region characteristic for rigid PUFs, although the PUF obtained from CNC-GL-EC polyol did not show the glass transition within −40 °C to 220 °C. Two maxima were observed on the dm/dT vs. temperature curve (Figure 6b) in the case of the sample of PUF obtained from HPC-derived polyol at 295 and 460 °C. The first peak was attributed to the decomposition of polyurethane to amine and carbon dioxide [40]; the second peak corresponds to methylene-phenyl bond dissociation (aromatic ring origin from pMDI [46,47].

In the case of PUF obtained from the CEL-H_2_O-GL-EC polyol, other endothermic peaks were observed at 260, 325, and 395–400 °C. The first one is related to the thermal dissociation of urethane and urea bonds; the second one relates to the conversion of amine and carbon dioxide as in other cases; and the third one is attributed to the dissociation of ether bonds, which are present in polyols. Also, the decomposition of cellulose mers gives a similar effect [46,47]. The peak at 310 °C was observed in the thermogram of the CNC-GL-EC polyol as a peak arising from overlapping peaks centered at 260 and 325 °C.

**Figure 7:**
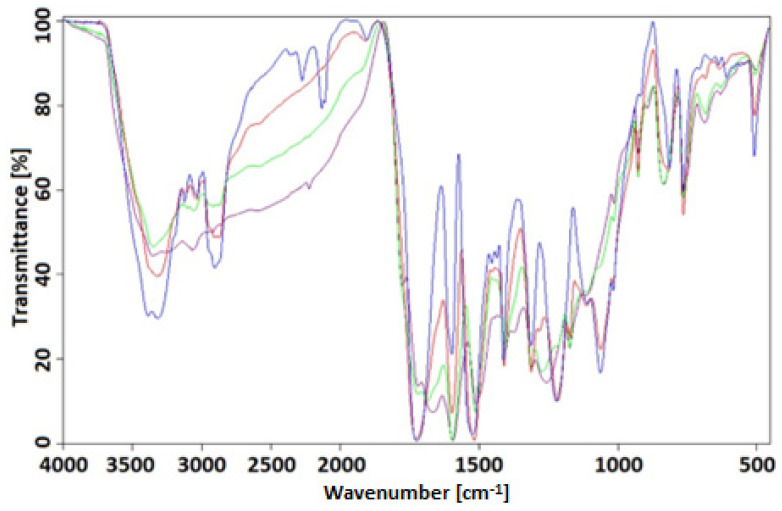
IR spectra of unheated (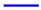) and annealed foam at 150 °C (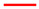), 175 °C (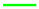), and 200 °C (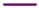) obtained from polyol CEL-H_2_O-GL-EC.

IR spectra of thermally converted PUFs provide some structural information on chemical changes accompanying thermal conversion. In the case of non-annealed PUFs (Figure 7), the bands at 3400 cm^−1^ from N-H stretching vibrations and the series of bands within 3000–2900 cm^−1^ from C-H bonds were visible. The isocyanate groups remained unreacted in the PUF manifest by the presence of the peak at 2276 cm^−1^, but for the case of carbodiimide bonds, a band at 2136 cm^−1^ was also observed. The thermal exposure of PUF at 150 °C was accompanied by the disappearance of carbodiimide groups due to oxidation and the disappearance of isocyanate groups due to their reaction with available hydroxyl groups from polyol. At the same time, the intensity of the C=O group stretching vibration at 1730 cm^−1^ diminished, presumably due to amide bond thermal degradation. In the IR spectra of annealed PUFs, the new band centered at 1610 cm^−1^ appeared, which was assigned to the C=C bond, corresponding to the graphitization of the PUF, which was also responsible for the increase in compressive strength.

**Figure 8:**
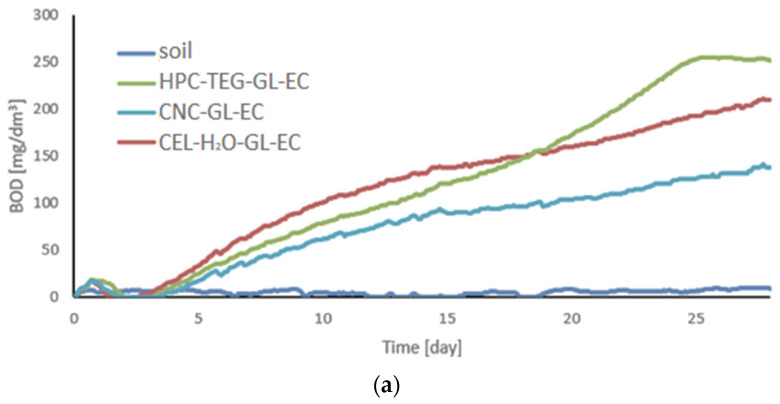

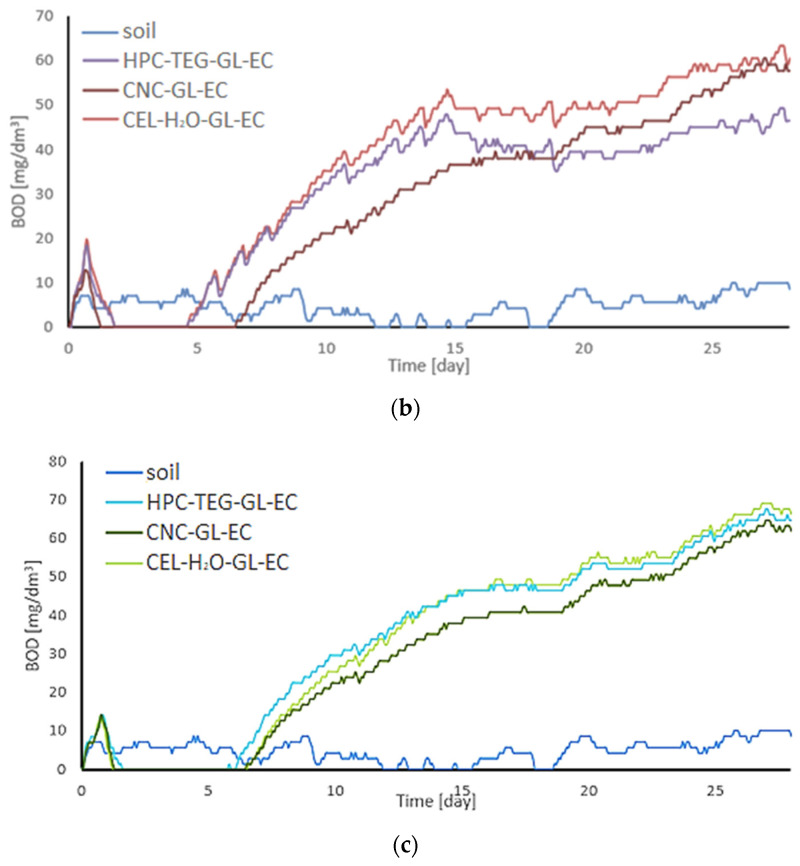
Results of biochemical oxygen demand of polyols (**a**) and foams in powder (**b**) and in the cube (**c**) during measurement.

Generally speaking, the severe worldwide problem is related to excessive amounts of plastic waste deposits. This problem is especially pronounced in the case of polyurethanes because they are very resistant to degradation. Therefore, it is necessary to search for biodegradable materials. We are focused on using cellulose as a substrate to obtain polyols, which are suitable for production polyurethanes from them. We tested the biodegradation of obtained polyols and PUFs. We applied the soil conditions to monitor the degradation of polyol, powdered PUF, and PUF-formed cubes and measured biological oxygen demand (BOD) in standard tests (Figure 8). The mass fractions of CHN elements were determined by the elemental analysis of polyol and PUF samples (Table 10) and used for theoretical predictions of BOD. 

The Dt of studied materials was determined after 28 days of soil biodegradation experiment, and the results are collected in Table 11.

We found that polyols degraded completely at this condition, while the degree of PUFs obtained from these polyols was 51–83%. We conclude that we obtained very promising materials, especially in the case of PUFs, which can be expected to decompose in soil within one year. We tested powdered PUFs according to the norm and then the cube of PUFs, which degraded faster than powder, which, as a result, may be an advantageous feature of the foam, as it does not require additional grinding before disposal.

**Figure 9:**
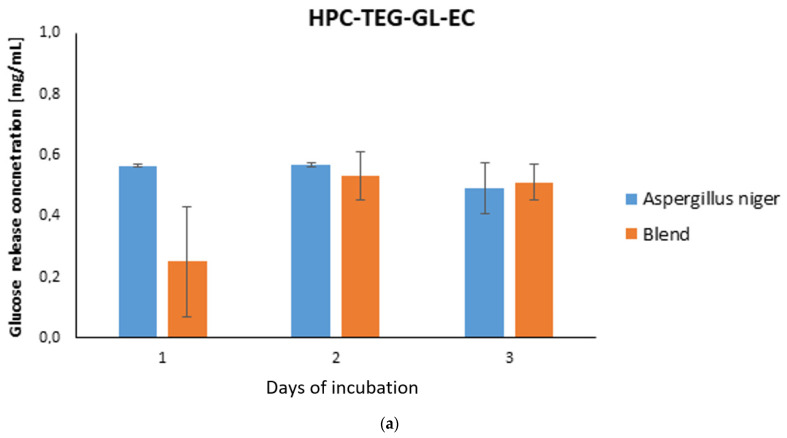

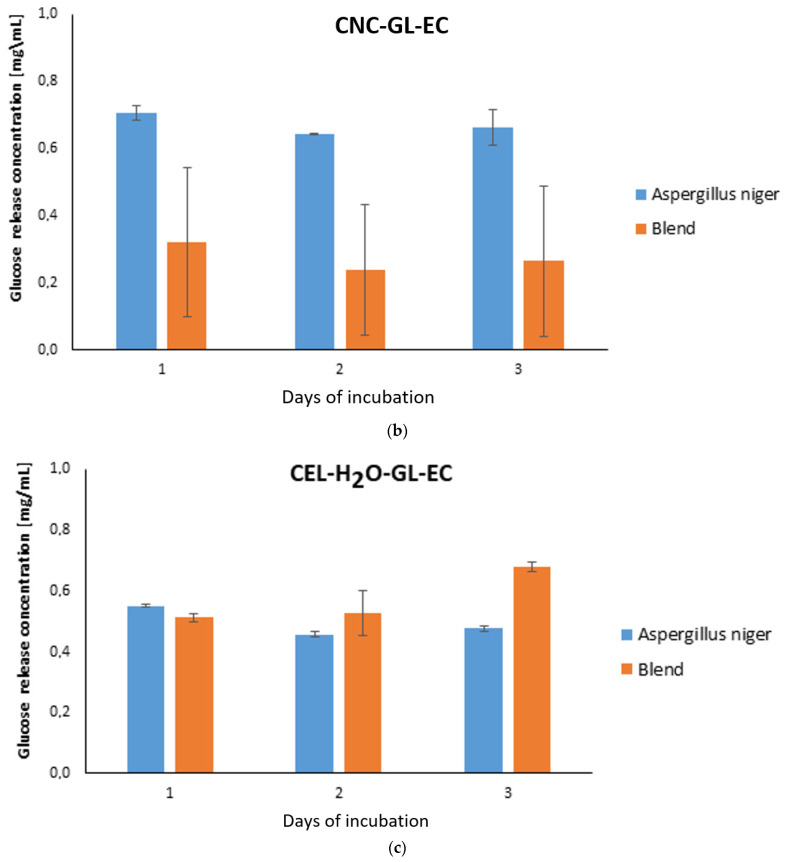
The glucose concentration after the cellulase treatment of the foams. The level of glucose was monitored with the DNS assay (details in the Materials and Methods) after 1, 2, or 3 days of incubation. (**a**) HPC-TEG-GL-EC; (**b**) CNC-GL-EC; (**c**) GEL-H_2_O-GL-EC.

**Figure 10:**
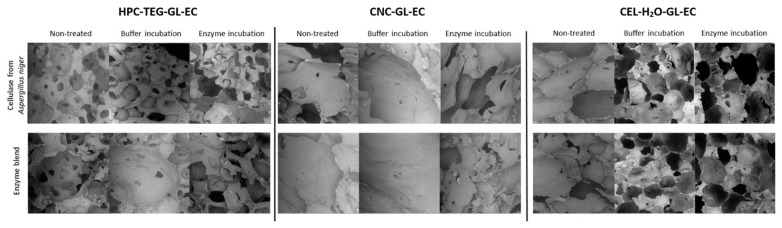
Representative SEM photomicrographs of the foams’ surface structure after enzyme incubation.

To confirm their biodegradation potential, we investigated whether the enzymes produced by different microbial strains were able to degrade foams and impact their structure. The microbial biodegradation of PUFs is linked to the production of enzymes, mostly hydrolases, which are inducible by the presence of the substrate. However, polyurethanes have been found to be susceptible to biodegradation by different microorganisms, albeit at a very low rate under environmental and laboratory conditions [48]. Synthesizing methods, chemical compounds, and ratios in the structure are the most important factors in the biodegradations of PUFs. The increasing soft segment ratio and biomaterial (cellulose, starch, etc.) content cause accelerated degradation. For enzymatic methods, biodegradation may occur by biological hydrolysis. Several studies on PUFs have already shown how urease, protease, and esterase enzymes are effective in biodegradation [49]. Here, we applied pure cellulase obtained from microorganisms to assess the degradation activity of one of the PUF compounds, cellulose. Among filamentous fungi, the *Aspergillus* genus is considered a model for cellulase production from bench to industrial scale [50]. Products obtained from the hydrolysis of a cellulose-containing substrate by cellulase consist of mainly glucose [51]. For the case of the capability of cellulase to cleave the polymeric structure of the tested foams, the increasing amount of glucose should be observed. Indeed, after the incubation of PUFs with cellulase obtained from *Aspergillus niger* or with a mixture of enzymes, a higher concentration of glucose is observed. However, the content of the glucose slightly depends on the type of PUFs tested. (Figure 9). These results may be attributed to the decomposition of the polysaccharide to a glucose monomer due to the fact that enzymes produce the cleavage of chains, making the polymer surface more susceptible to further degradation [52]. Based on these results, we analyzed the structure of the PUFs treated with cellulase purified from *Aspergillus niger* or a mixture of enzymes. Representative SEM images demonstrated the physical degradation only for PUFs incubated with the enzymes (Figure 10). The surface showed pits, cracking, and erosion, which could be attributed to enzyme activity.

Simultaneously, the samples not incubated or used after incubation but without an enzyme show no changes in the structure. Indeed, there are data reported on *Aspergillus* sp. and its ability to use PUF as a source of carbon and energy [53]. Moreover, Ozsagıroglu et al. reported on the effects of enzyme types and their mixtures on polymer biodegradation. Protease-type enzymes were more effective than esterase enzymes, while esterase had the potential to decompose polyurethane when it formed a mixture with protease [54]. A high specific surface area provides efficient contact for adsorbed enzymes or microorganisms and is an important factor in accelerating biodegradation. Also, PUF or cellulose particle size affects the surface area and bioavailability of PUF in the environment [55]. The bulk PUF can be then micronized to obtain a PUF powder with a particle size of tens of microns, which makes the sample bioavailable.

## 4. Conclusions

The structure and physical properties of polyols obtained from (hydroxypropyl)cellulose, hydrolyzed cellulose, and cellulose were determined, including density, viscosity, and surface tension. The polyol obtained from (hydroxypropyl)cellulose in the triethylene glycol solvent was accompanied by oligomeric products of glycol hydroxyalkylation and oligomeric glycidol. The polyol obtained by the hydroxyalkylation of cellulose with glycidol and ethylene carbonate in the water contained mers of hydroxyalkylated cellulose and products of the hydroxyalkylation of water.

Polyurethane foams obtained from cellulose-based polyols are typical rigid foams with beneficial thermal resistance, enabling us to use such materials at temperatures elevated up to 150 °C, or in some cases, to 175 °C.

All the obtained materials are environmentally friendly; polyols are fast and biodegradable, while the polyurethane foams obtained from them are degraded up to 51 to 83% after 28 days of soil degradation at ambient temperature, according to the BOD test.

Different types of PUFs were biodegradable by cellulase, most effectively by an enzyme isolated from *Aspergillus* sp.

## Figures and Tables

**Table 1:** Interpretation of MALDI-ToF spectrum of polyol obtained from CNC, GL, and EC.

Entry	Signal Position *m*/*z*	Relative Intensity of Signal [%]	The Molecular Ion Structure	Calc. Molecular Weight [g/mol]
1	87.048	14.6	EC	88.016
2	140.888	12.5	GL + OE + Na^+^	141.053
3	155.987	16.7	GL + OE + K^+^	157.027
4	173.061	11.5	2 GL + OE − H_2_O	174.089
5	196.984	71.9	2GL + EO − H_2_O + Na^+^	197.079
6	217.103	11.5	2GL + 2EO − H_2_O	218.115
7	245.101	9.4	GL + 3OE + K^+^	245.079
8	261.131	9.2	2GL + 3EO − H_2_O	262.142
9	289.130	19.8	GL + 4OE + K^+^	289.105
10	303.132	30.2	2GL + 3EO + Na^+^	305.142
11	319.096	14.6	2GL + 3OE + K^+^	319.116
12	333.133	36.5	GL + 5OE + K^+^	333.132
13	347.151	25.0	2GL + 4OE + Na^+^	347.168
14	363.130	18.8	2GL + 4OE + K^+^	363.142
15	377.169	29.2	GL + 6OE + K^+^	377.158
16	393.985	100	3GL + 3OE + K^+^	393.153
17	407.147	16.7	2GL + 5OE + K^+^	407.168
18	421.169	18.8	GL +7OE + K^+^	421.184
19	437.157	16.6	3GL + 4OE + K^+^	437.179
20	451.186	15.6	2GL + 6OE + K^+^	451.195
21	465.193	12.5	GL + 8OE + K^+^	465.598
22	481.179	13.5	3GL + 5OE + K^+^	481.205
23	495.208	12.3	2GL + 7OE + K^+^	495.221
24	507.211	11.5	3GL + 6OE − H_2_O + K^+^	507.221
25	525.207	12.3	3GL + 6OE + K^+^	525.231
26	539.224	11.5	2GL + 8OE + K^+^	539.247
27	555.221	8.3	4GL + 5OE + K^+^	555.242
28	569.248	10.4	3GL + 7OE + K^+^	569.258
29	581.235	9.4	4GL + 6OE − H_2_O + K^+^	581.258
30	590.878	75.0	5GL + 5EO	590.315
31	613.280	8.4	3GL + 8OE + K^+^	613.284
32	625.296	7.9	4GL + 7OE − H_2_O + K^+^	625.284
33	643.281	10.8	4GL + 7OE + K	643.294
34	687.296	5.2	4GL + 8OE + K	687.321

Description of abbreviations used in the table: GL—glycidol; OE—oxyethylene group; Na^+^—sodium cation; K^+^—potassium cation; -H_2_O—means dehydration of the product.

**Table 2:** Interpretation of MALDI-ToF spectrum of polyol obtained from CEL, GL, and EC in aqueous conditions.

Entry	Signal Position (*m*/*z*)	Relative Intensity of Signal [%]	The Molecular Ion Structure	Calc. Molecular Weight [g/mol]
1.	102.078	100.0	H_2_O + OE + K^+^	101.001
2.	221.802	10.4	3GL	222.110
3.	265.806	69.2	3 GL + OE	266.137
4.	322.935	7.7	H_2_O + 3GL + OE + K^+^	323.111
5.	336.944	4.3	H_2_O + 2GL + 3OE + K^+^	337.127
6.	352.948	3.4	H_2_O + 4GL + K^+^ GL + 5OE + K^+^	353.121 353.288
7.	366.947	9.0	H_2_O + 3GL + 2OE + K^+^	367.137
8.	396.944	10.6	H_2_O + 4GL + OE + K^+^ GL + 6OE + K^+^	397.148 397.314
9.	410.939	6.5	H_2_O + 3GL + 3OE + K^+^	411.163
10.	426.928	4.5	2GL + 5OE + K^+^ H_2_O + 5GL + K^+^	427.325 427.158
11.	440.946	11.6	H_2_O + 4GL + 2OE + K^+^	441.174
12.	470.939	13.5	2GL + 6OE + K^+^ H_2_O + 5GL + OE + K^+^	471.351 471.184
13.	484.94	8.2	GL + 8OE + K^+^ H_2_O + 4GL + 3OE + K^+^	485.367 485.200
14.	514.934	13.9	2GL + 7OE + K^+^ H_2_O + 5GL + 2OE + K^+^	515.377 515.211
15.	544.937	14.1	3GL + 6OE + K^+^ H_2_O + 6GL + OE + K^+^	545.388 545.221
16.	558.956	8.9	2GL + 8OE + K^+^ H_2_O + 5GL + 3OE + K^+^	559.404 559.237
17.	588.956	13.6	3GL + 7OE + K^+^ H_2_O + 6GL + 2OE + K^+^	589.414 589.247
18.	618.954	11.9	4GL + 6OE + K^+^ H_2_O + 7GL + OE + K^+^	619.425 619.258
19.	632.962	9.2	3GL + 8OE + K^+^ H_2_O + 6GL + 3OE + K^+^	633.44 633.274
20.	662.964	11.1	4GL + 7OE + K^+^ H_2_O + 7GL + 2OE + K^+^	663.451 663.284
21.	691.965	7.1	4GL + 9OE	692.383
22.	736.992	5.9	5GL + 7OE + K^+^ H_2_O + 8GL + 2OE + K^+^	737.488 737.321
23.	766.984	3.3	6GL + 6OE + K^+^ H_2_O + 9GL + OE + K^+^	767.498 767.332
24.	811.008	2.8	6GL + 7OE + K^+^ H_2_O + 9GL + 2OE + K^+^	811.524 811.358
25.	825.027	1.5	H_2_O + 8GL + 4OE + K^+^	825.373
26.	840.985	1.5	7GL + 6OE + K^+^ H_2_O + 10GL + OE + K^+^ 6GL + 9OE	841.535 841.368 840.457
27.	855.005	1.8	6GL + 8OE + K^+^ H_2_O + 9GL + 3OE + K^+^	855.551 855.384
28.	884.977	1.8	7GL + 7OE + K^+^ H_2_O + 10GL + 2OE + K^+^	885.175 885.395
29.	908.585	1.5	H_2_O + 2GL + 16OE + K^+^ 7GL + 8OE + K^+^	909.467 909.431
30.	928.995	1.1	7GL + 8OE + K^+^ H_2_O + 10GL + 3OE + K^+^	929.587 929.421
31.	958.991	1.0	8GL + 7OE + K^+^ H_2_O + 11GL + 2OE + K^+^	959.598 959.431

Description of abbreviations used in the table: GL—glycidol; OE—oxyethylene group from EC; H_2_O—water; K^+^—ion K^+^ from the catalyst K_2_CO_3._

**Table 3:** Molecular weights and dispersity of polyols.

Polyol	Fraction Content [%]	*M*_n_ [u]	*M*_w_ [u]	*M*_z_ [u]	*M*_w_/*M*_n_
HPC-TEG-GL-EC	100	1365	1969	3891	1.44
	7.08	99,860	155,401	209,810	1.56
	92.92	1341	1906	2911	1.42
CNC-GL-EC	100	2514	13,557	40,439	5.39
	61.81	9855	21,081	42,221	2.14
	38.19	1125	1473	1791	1.31
CEL-H_2_O-GL-EC	100	1650	2676	4202	1.62

**Table 4:** Physical properties of polyols at 20 °C.

Polyol	Density [g/cm^3^]	Viscosity [mPa·s]	Surface Tension [N/m·10^−3^]	Hydroksyl Number [mg KOH/g]
HPC-TEG-GL-EC	1.190	379,127	50.96	481
CNC-GL-WE	1.300	42,325	52.92	473
CEL-H_2_O-GL-EC	1.283	5538	49.00	688

**Table 5:** The influence of composition on the foaming process.

Polyol	Composition [g/100 g of Polyol]				IC	Foaming Process		
	pMDI	Water [%]	TEA	Silicon L-6900		Cream Time [s]	Growing Time [s]	Tack Free Time [s]
HPC-TEG-GL-EC	175	2	0.2	8.8	1.5	61	35	1
CNC-GL-EC	120	2	1.3	3.9	1.0	32	32	1
CEL-H_2_O-GL-EC	160	2	0.3	3.1	1.0	129	157	1

Cream time—the time elapsed from the moment of mixing to the start of volume expansion; growing time—the time from the start of expansion to the moment of reaching the sample final volume; tack free time—the time from the sample reaching its final volume to the moment of losing its surface adhesion to powdered substances; IC—isocyanate coefficient.

**Table 6:** Some properties of polyurethane foams.

Foam Obtained from Polyol	Density [kg/m^3^]	Absorption of Water [wt%] After			Thermal Conductivity Coefficient [W/m·K]	Dimensional Stability [%] in Temperature 150 °C					
		5 min	3 h	24 h		Length Change After		Width Change After		Hight Change After	
						20 h	40 h	20 h	40 h	20 h	40 h
HPC-TEG-GL-EC	58.1	0.25	0.89	2.12	0.0354	+3.13	+4.56	−5.07	−4.16	−3.53	−2.89
CNC-GL-EC	72.9	1.47	3.12	4.94	0.0364	−0.67	−0.80	−3.36	−4.00	−1.37	−1.51
CEL-H_2_O-GL-EC	60.5	4.47	5.40	6.56	0.0338	−0.69	−0.44	−1.45	0.27	−2.52	−3.06

**Table 7:** Results of micrograph analyses.

Foam Obtained from Polyol	Large Diameter [µm]	Smaller Diameter [µm]
HPC-TEG-GL-EC	140 ± 14	90 ± 13
CNC-GL-EC	290 ± 20	170 ± 16
CEL-H_2_O-GL-EC	140 ± 15	110 ± 14

**Table 8:** Mass loss and compressive strength of polyurethane foams after one month of exposure to high temperature.

Polyetherol	Mass Loss [wt%]. After Month Exposure in Temperature [°C]			Compressive Strength [MPa]			
				Before Exposure	After Exposure in Temperature [°C]		
	150	175	200		150	175	200
HPC-TEG-GL-EC	9.83	29.27	44.48	0.279	0.350	0.549	0.725
CNC-GL-EC	7.57	20.97	-	0.212	0.325	0.410	-
CEL-H_2_O-GL-EC	13.00	30.67	46.15	0.234	0.284	0.379	0.519

Mass loss was determined with ±0.55% accuracy. Compressive strength measured at 10% deformation. Compression strength was determined with 2.5% accuracy.

**Table 9:** Thermal analysis of foam determined by dynamic analysis.

Polyetherol	T_5%_ [°C]	T_10%_ [°C]	T_25%_ [°C]	T_50%_ [°C]	T_max_ of Decomposition [°C]	T_g_ [°C]
HPC-TEG-GL-EC	269	280	304	371	293	92
CNC-GL-EC	274	293	326	380	328	-
CEL-H_2_O-GL-EC	245	263	314	390	397	97

**Table 10:** Mass fractions of individual elements in polyols and the obtained polyurethane foam.

Material	Element			
	C	H	O	N
Polyol HPC-TEG-GL-EC	0.4761	0.0912	0.4327	0
Polyol CNC-GL-EC	0.4970	0.0845	0.4185	0
Polyol CEL-H_2_O-GL-EC	0.4530	0.0867	0.4603	0
Foam (powder) from HPC-TEG-GL-EC	0.6385	0.0613	0.2335	0.0667
Foam (powder) from CNC-GL-EC	0.6237	0.0610	0.2514	0.0639
Foam (powder) from CEL-H_2_O-GL-EC	0.6182	0.0603	0.2563	0.0652
Foam (cube) from HPC-TEG-GL-EC	0.6385	0.0613	0.2335	0.0667
Foam (cube) from CNC-GL-EC	0.6237	0.0610	0.2514	0.0639
Foam (cube) from CEL-H_2_O-GL-EC	0.6182	0.0603	0.2563	0.0652

**Table 11:** Results of susceptibility to the biodegradation of polyols and foams.

Material	BODx [mg/L]	BOD_28_ [mg/L]	Sample Mass [g]	TOD Counts	TOD [mg/L]	Dt [%]
Polyol HPC-TEG-GL-EC	251.0	242.5	0.24	9.0416	37.67	100
Polyol CNC-GL-EC	138.0	129.5	0.21	9.8840	47.07	100
Polyol CEL-H_2_O-GL-EC	209.0	200.5	0.19	7.8248	41.18	100
PUF (powder) HPC-TEG-GL-EC	46.5	38.0	0.21	15.5856	74.22	51.2
PUF (powder) CNC-GL-EC	57.8	49.3	0.20	14.8904	74.45	66.2
PUF (powder) CEL-H_2_O-GL-EC	60.6	52.1	0.20	14.5992	73.00	71.4
PUF (cube) HPC-TEG-GL-EC	64.8	56.3	0.21	15.5856	74.22	75.9
PUF (cube) CNC-GL-EC	62.0	53.5	0.20	14.8904	74.45	71.9
PUF (cube) CEL-H2O-GL-EC	66.2	57.7	0.21	14.5992	69.52	83.0

## Data Availability

The raw data supporting the conclusions of this article will be made available by the authors on request.
